# Risk factors and outcomes of hyperactive delirium in older medical inpatients admitted to non-intensive care unit: a prospective cohort study

**DOI:** 10.1186/s12888-025-06731-5

**Published:** 2025-04-03

**Authors:** Panumas Kaewpongsa, Kulapong Jayanama, Sirasa Ruangritchankul

**Affiliations:** 1 Department of Medicine, Faculty of Medicine Ramathibodi Hospital, Mahidol University, Bangkok, Thailand; 2 Faculty of Medicine Ramathibodi Hospital, Chakri Naruebodindra Medical Institute, Mahidol University, Samut Prakan, Thailand; 3 Division of Geriatric Medicine, Department of Medicine, Faculty of Medicine Ramathibodi Hospital, Mahidol University, Bangkok, Thailand

**Keywords:** Hyperactive delirium, Older adults, Risk factor, Mortality

## Abstract

**Background:**

Hyperactive delirium is a common complication in older medical inpatients in non-intensive care units. This condition increases the risk of diminished physical function, morbidity, and mortality. Moreover, antipsychotics and sedatives were widely used in these patients, contributing to many drug interactions and adverse drug reactions. This study aimed to evaluate the risk factors for hyperactive delirium and assess adverse outcomes among these susceptible patients.

**Methods:**

We conducted a prospective observational study to examine hyperactive delirium as an exposure and its association with adverse outcomes without intervention. A total of 238 medical patients aged ≥ 60 admitted to non-intensive care units at Ramathibodi Hospital between September 1, 2022, and December 31, 2023, were enrolled. The clinical characteristics, physical examination, and biochemical profiles at baseline were assessed. Adverse clinical outcomes at 90 days after discharge were evaluated by reviewing electronic medical records (EMRs). The Confusion Assessment Method and Richmond Agitation-Sedation Scale (RASS) score of + 1 to + 4 were used to diagnose hyperactive delirium. The Cox proportional hazard model was performed to identify risk factors and adverse clinical outcomes associated with hyperactive delirium, with results reported as hazard ratios (HRs) and 95% confidence intervals (CIs).

**Results:**

Overall, hyperactive delirium was diagnosed in 115 (48.3%) patients and had an incidence rate of 101.1 cases per 1000 person-days. The risk factors for hyperactive delirium were urinary incontinence (HR 1.69, 95% CI 1.11–2.57), clinical frailty scale (CFS) ≥ 5 (HR 2.79, 95% CI 1.69–4.62), and Montreal Cognitive Assessment (MoCA) score < 25 (HR 4.63, 95% CI 1.09–19.75). Within 90 days after discharge, 14 (12.2%) patients with delirium had died. Medical inpatients who experienced hyperactive delirium had an 8.23-fold increased risk of 90-day mortality following hospital discharge compared to those without delirium (HR 8.23, 95% CI 1.38–48.98).

**Conclusions:**

The risk factors for hyperactive delirium were urinary incontinence, frailty (CFS score ≥ 5), and cognitive impairment (MoCA score < 25). Among older medical inpatients, hyperactive delirium was an independent predictor of 90-day mortality after discharge.

**Supplementary Information:**

The online version contains supplementary material available at 10.1186/s12888-025-06731-5.

## Introduction

With rising longevity, the aging population has increased markedly in recent decades [1]. In Thailand, the population aged ≥ 60 was estimated at 13.3 million in 2021 and is predicted to be 16.7 million by 2040 [2]. Several geriatric individuals tend to need more medical treatment and hospitalization owing to the increased incidence of chronic diseases and geriatric syndromes [3]. According to a previous study [4], older adults accounted for 65% of admitted patients and occupied 70% of hospital days. Delirium is a common complication among hospitalized older patients, with a reported incidence ranging from 11 to 66%, depending on the healthcare setting and screening tools [5–7]. Delirium is a clinical and multifactorial syndrome of attention deficit and cognitive disorder [8]. As a multifactorial syndrome, the risk factors for delirium comprise a complex interrelationship among predisposing and predisposing factors. Approximately 30%−40% of these factors are preventable [9]. A previous report identified an association between depression and delirium [10]. In addition, the concurrent use of five or more medications, particularly antipsychotic polypharmacy, was designated a risk factor for delirium [11, 12]. Therefore, prudent medication selection is necessary to avoid interactions and delirium as an anticholinergic effect.

The Health Belief Model (HBM) is a widely recognized theoretical framework employed to evaluate the risk of health conditions, including delirium [13]. Additionally, HBM serves as a guiding model to promote patient engagement in proactive health behaviors to prevent deterioration in health status.

For several decades, various diagnostic criteria for delirium have been developed and widely applied in the medical practice, including the Diagnostic and Statistical Manual of Mental Disorders, fifth edition (DSM-V) [8], the Confusion Assessment Method (CAM) [14], and the 4 A’s test (4AT) [15]. According to the DSM-V criteria, delirium is categorized into three subtypes of psychomotor symptoms: hypoactive, hyperactive, and mixed types of activity [16]. Hyperactive delirium contributes to worsening clinical prognosis, including falls and death, and increases healthcare expenditures [17]. Moreover, psychotropics are often prescribed for controlling agitation and aggression symptoms of hyperactive delirium, leading to many drug interactions and adverse drug events [18]. Although several studies have explored older cases of delirium in Thailand, specific knowledge of hyperactive delirium and its adverse clinical outcomes in older medical inpatients admitted to non-intensive care units (ICU) remains limited. Therefore, we aimed to determine the risk factors and adverse clinical outcomes of hyperactive delirium in this susceptible population.

## Material and Methods

### Research hypothesis and objectives

This study examined the null hypothesis that no significant difference exists in the rate of unexpected rehospitalization, unplanned emergency department (ED) visits, and mortality based on the presence of hyperactive delirium. Using the PICO approach [19], the research hypothesis was formulated as follows: older medical inpatients admitted to non-ICU settings at Ramathibodi Hospital, Mahidol University (Population) who experienced hyperactive delirium (Intervention) were compared to those without delirium (Comparison) in related to unexpected rehospitalization, unplanned ED visits, and mortality (Outcomes). The primary objective was to evaluate 90-day adverse outcomes after discharge among older medical inpatients admitted to non-ICU settings. Additionally, the secondary objective aimed to identify risk factors associated with hyperactive delirium in this high-risk population.

### Study design, setting and participants

This prospective observational study was conducted to investigate the association between hyperactive delirium as an exposure and adverse outcomes, such as unexpected rehospitalizations, unplanned ED visits, and mortality, without any intervention. The study consecutively enrolled 238 older medical patients aged ≥ 60 admitted to non-intensive care units at Ramathibodi Hospital, Mahidol University from September 1, 2022 to December 31, 2023. During hospitalization, all participants were closely monitored for hyperactive delirium, and their conditions were recorded daily by trained medical staff from admission until the onset of hyperactive delirium, transfer, death, or discharge. Furthermore, adverse clinical outcomes were tracked at 30 and 90 days post-discharge by reviewing electronic medical records (EMRs). All eligible patients were anticipated to have a length of hospital stay longer than 48 h. Patients were excluded if they were unable to communicate and provide informed consent because of being comatose, having severe dementia, blindness, or severe deafness. Additionally, patients diagnosed with delirium or treated with antipsychotics or benzodiazepines for delirium on admission, those with a terminal illness with a life expectancy of less than 3 months, or a history of severe psychiatric problems were excluded from our study. Older medical inpatients who developed hypoactive delirium or mixed type were also excluded from this study. Hospitalized patients who were subsequently transferred to the intensive care unit or died in the hospital were also excluded from the analysis.

**Fig. 1:**
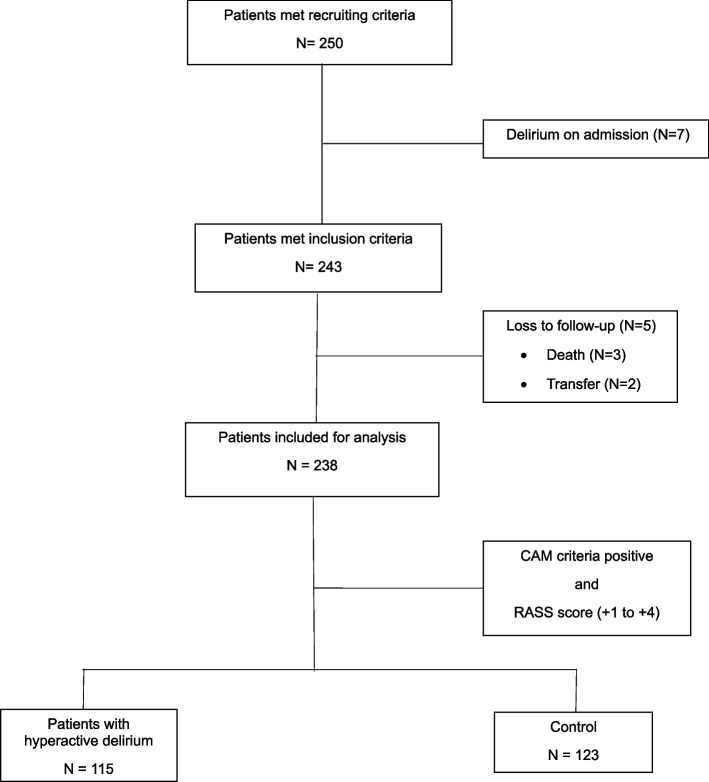
Flow chart of sample recruitment

The sample size was calculated using the n4studies sample size application, Thailand [20]. According to a previous report on the association between depression and delirium [10] with type I error of 0.05, type II error of 0.2, exposed proportion of 0.47, and unexposed proportion of 0.65, the calculated sample size was 238. The study recruited 250 older patients admitted to non-ICU internal medicine wards during the inclusion period. Of these, 7 were excluded because of delirium on admission, and 5 patients were lost to follow-up because of transfer to intensive care units or hospital death. The remaining 238 hospitalized older patients were included in the data analysis, as shown in Fig. 1. The CAM and Richmond Agitation Sedation Scale (RASS) score [21] of + 1 to + 4 were used to diagnose hyperactive delirium and categorize the patients into 115 patients with hyperactive delirium and 123 patients without delirium (control group).

### Data collection and measurement tools

The collected data were provided from eligible participants admitted to the internal medicine wards (non-ICU) at Ramathibodi Hospital, Mahidol University from September 1, 2022 to December 31, 2023. Within 48 h of admission, trained medical researchers gathered comprehensive data from EMRs, interview-administered questionnaires, clinical and functional assessments, and biochemical profiles of the eligible patients. The 238 participants included in the analysis completed the clinical and functional assessments and interview-administered questionnaire. The EMR data included demographic characteristics, such as sex and age; health conditions, including the primary diagnosis, chronic medical diseases and psychiatric problems; body weight and height; currently prescribed medications; healthcare services, such as length of hospital stay (LOS), history of ED and outpatient department visits, and hospital admission within the past 12 months; and hyperactive delirium-related biochemical parameters. The list and number of all prescribed medications before the onset of hyperactive delirium were recorded. Medical assessors evaluated benzodiazepines, narcotic analgesics, anticholinergics, and corticosteroids as high-risk drugs for delirium [22].

A face-to-face interview questionnaire was administered to gather information on socio-demographic profiles, geriatric syndrome, psycho-cognitive status, and functional capacity. Trained assessors performed clinical measurements of body temperature, pulse rate (PR), oxygen saturation, systolic blood pressure (SBP), diastolic blood pressure (DBP), clinical frailty, nutritional status, weight, height, and body mass index (BMI). Moreover, physical restraints and medical devices, such as urinary catheters, peripheral and central venous catheters, non-invasive mechanical ventilation (NIMV), drains, oxygen therapy, and nasogastric tube, were recorded.

### Face-to-face interview-administered questionnaire

A preliminary version of the questionnaire was initially administered as a pilot interview. Ten inpatients, who are representative of the target population, participated to provide feedback for refining the questionnaire. A total of 238 eligible older medical inpatients admitted to non-ICU settings were interviewed by trained medical personnel. The revised questionnaire was administered within 48 h of admission to assess sociodemographic profiles, including educational level, need for caregivers, living and marital status, and medical insurance; lifestyles, such as smoking, alcoholic drinking, and herbal and substance use; and the geriatric syndromes such as visual impairment, pressure ulcer, or fall, which are conditions representing complex clinical impairment in older adults, as shown in Supplementary Assessment 1. The presence of geriatric syndrome was evaluated using the binary question “Have you had a pressure ulcer?” with an answer of “yes” encoded as 1. Functional status was evaluated using Barthel activity of daily living (ADL) index [23] and Lawton Instrumental ADL (IADL) index [24]. The former assessed fundamental skills on ambulation, dressing, bathing, feeding, self-grooming, toileting, and incontinence, ranging from 0 (highest level of dependence) to 20 (least level of dependence) [23], whereas the latter measured eight domains of complex activities on telephone use, transportation, food preparation, shopping, managing finances, handling medications, laundering, and housework [24]. The total scores were scaled from 0 to 8, with higher scores indicating greater functional capacity. Cognitive function was evaluated using the Montreal Cognitive Assessment (MoCA), which measured higher cortical functions, including language, memory, executive functions, visuoconstructional skills, abstract reasoning, attention, and calculation [25, 26]. The total MoCA score ranged from 0 to 30, with lower scores reflecting worse cognitive function and a total score of MoCA < 25 representing cognitive impairment in the Thai population. In addition, trained personnel assessed the presence of depression using the Geriatric Depression Scale-15 (GDS-15), which ranged from 0 to 15. The GDS-15 score of > 5 was considered depression [27].

### Clinical assessments

On admission, the body weight, height, and vital signs, including SBP, DBP, PR, oxygen saturation, and body temperature, were measured in all patients. Experienced staff evaluated the risk of malnutrition using a Nutritional Alert Form (NAF), which comprised eight sections of BMI (kg/m^2^) [28], body build, dietary intake and pattern, weight change, chewing and swallowing problems, medical conditions related to malnutrition, functional capacity, serum albumin, and lymphocyte count. The Severity of malnutrition was classified according to the NAF score as follows: normal-mild (0–5), moderate (6–10), and severe (≥ 11) [29]. Additionally, the patients were characterized as frail according to the Clinical Frailty Scale (CFS) [30], which ranged from 1 (very fit) to 9 (terminally ill) [31]. The patients with a CFS score of 5 or higher were considered frail [30].

### Definitions

Delirium was defined as an acute onset neuropsychiatric syndrome, which was characterized by a fluctuating course of disrupted attention and altered levels of cognitive function and consciousness [32]. Patients with motor agitation, aggression, and restlessness were considered to have hyperactive delirium [16], which was confirmed by the CAM and RASS scores.

### Delirium assessment

The CAM algorithm is a validated bedside screening tool for delirium, assessing four key features: acute onset with a fluctuating course, inattention, disorganized thinking, and altered level of consciousness [14]. A diagnosis of delirium is considered positive when both acute onset with a fluctuating course and inattention are present, along with either disorganized thinking or an altered level of consciousness. This operationalized instrument was easy to apply and was reported to have high sensitivity (90%) and specificity (94%) [33]. After the diagnosis of delirium by the CAM criteria, a RASS score of + 1 to + 4 was further used to identify hyperactive delirium [21]. The CAM and RASS scores were directly observed at the bedside and recorded daily by trained medical personnel from admission until the development of hyperactive delirium, transfer, death, or hospital discharge. The time to onset of hyperactive delirium after hospital admission was collected for analysis.

### Outcomes

The primary outcomes were adverse clinical outcomes of hyperactive delirium after discharge. Unexpected hospital admission, unplanned ED visits, and all-cause mortality at 30 and 90 days after discharge were evaluated by follow-up EMR review. The secondary outcomes were new-onset hyperactive delirium and its risk factors.

### Statistical analysis

The statistical analyses were performed using the SPSS for Windows Software Package, Version 25 (SPSS Inc., Chicago, Ill., USA). The baseline clinical characteristics and biochemical profiles were described as mean ± standard deviation (SD) or median ± interquartile range (IQR) for continuous variables and as number (percentage) for categorical variables. The data were compared between the two groups using Pearson’s chi-square test or Fisher’s exact test for categorical variables and Unpaired Student’s t-test or Mann–Whitney U test for continuous variables. Multivariate Cox proportional hazard models were used to evaluate the risk factors for hyperactive delirium. Furthermore, the rates of mortality and unplanned ED visits, and hospital readmission at 90 days after discharge were compared between the two groups. The Cox proportional hazard model adjusted for MoCA score, NAF score, CFS score, age, sex, number of comorbidities, and polypharmacy was used to determine the potential effect of hyperactive delirium on these outcomes. The results were presented as hazard ratios (HRs) and 95% confidence interval (CI). Statistical significance was set at a level of *p* < 0.05.

#### Ethical approval declarations

The study was approved by the Committee on Human Rights Related to Research Involving Human Subjects, Faculty of Medicine Ramathibodi Hospital, Mahidol University (protocol number: COA. MURA 2022/433) on 27th July 2022. All methods were in compliance with the relevant guidelines and regulations in the Declaration of Helsinki. This prospective observational study was conducted and reported in accordance with the guideline of the Strengthening the Reporting of Observational Studies in Epidemiology (STROBE) statement.

## Results

### Baseline clinical characteristics and biochemical parameters

The hyperactive delirium group comprised 115 patients (48.3%), and the non-delirium or control group comprised 123 patients (51.7%). The overall mean age was 74.9 (SD 8.1), ranging from 60 to 94 years. The descriptive and comparative analyses of the baseline characteristics of the two groups are revealed in Table 1. Patients with hyperactive delirium were more likely to be 65 years and over (*p* < 0.001) and to have an educational level of ≤ 12 years (*p* = 0.008) compared to patients without delirium. Patients in both groups had similar lifestyles (number of smokers, alcohol drinkers, and herbal users), with *p* > 0.05. A substantially lower proportion of patients with hyperactive delirium were able to take care of themselves than those in another group (*p* < 0.001). Additionally, patients with hyperactive delirium had significantly longer LOS than those without delirium [7 days (IQR 4–12.8) vs. 5 days (IQR 3–8), *p* = 0.002].

**Table 1:** Baseline demographics among older medical inpatients stratified by exposure to hyperactive delirium

Baseline characteristics	All (*n* = 238)	Hyperactive delirium (*n* = 115)	Control (*n* = 123)	*P-*value
		**N (%)**	**N (%)**	
**Age (years)**, mean (SD)	74.9 (8.1)	78.1 (7.6)	71.9 (7.5)	< 0.001^#^
< 65 years	29 (12.2)	3 (2.6)	26 (21.1)	< 0.001^*^
≥ 65 years	209 (87.8)	112 (97.4)	97 (78.9)	
Female	140 (58.8)	74 (64.3)	66 (53.7)	0.094^*^
**Educational status (years)**				
≤ 12	176 (73.9)	94 (81.7)	82 (66.7)	0.008^*^
> 12	62 (26.1)	21 (18.3)	41 (33.3)	
**Marital status**				
Single	21 (8.8)	7 (6.1)	14 (11.4)	0.150^*^
Married, widowed, or separated	217 (91.2)	108 (93.9)	109 (88.6)	
**Lifestyle**				
Drinking	46 (19.3)	19 (16.5)	27 (22.0)	0.289^*^
Smoking	67 (28.2)	29 (25.2)	38 (30.9)	0.330^*^
Herbal use	7 (2.9)	1 (0.9)	6 (4.9)	0.121^*^
**Physical self-care**				
Self-care	184 (77.3)	68 (59.1)	116 (94.3)	< 0.001^*^
Self-drug administration	194 (81.5)	80 (69.6)	114 (92.7)	< 0.001^*^
**Healthcare services**				
Paying for health care; self-paid	77 (32.4)	39 (33.9)	38 (30.9)	0.619^*^
History of OPD visits > 10 within 1 year	129 (54.2)	69 (60.0)	60 (48.8)	0.083^*^
History of hospital admission > 1 within 1 year	24 (10.1)	13 (11.3)	11 (8.9)	0.545^*^
History of ER visit > 1 within 1 year	58 (24.4)	34 (29.6)	24 (19.5)	0.071^*^
LOS, median (IQR)	5 (3, 9.3)	7 (4, 12.8)	5 (3, 8)	0.002^+^

Data are presented as mean (standard deviation), N (%), or median (interquartile range)

*Abbreviations: SD* standard deviation, *IQR* interquartile range, *ER* emergency room, *OPD* outpatient department, *LOS* length of stay

^*^Chi-square test

^#^ Student’s t-test

^+^ Mann–Whitney U test

The three most common chronic diseases in both groups were hypertension (84.5%), dyslipidemia (83.6%) and anemia (49.2%). There was no difference in the number of chronic diseases between the two groups (*p* > 0.05), except the likelihood of congestive heart failure (CHF) that was higher in the hyperactive delirium group than in another group (*p* = 0.045), as shown in Table 2. In the hyperactive delirium group, the most common leading cause of hospital admission was CHF (20.0%).

**Table 2:** Clinical characteristics among older medical inpatients stratified by exposure to hyperactive delirium

Clinical characteristics	All (*n* = 238)	Hyperactive delirium (*n* = 115)	Control (*n* = 123)	*P-*value
		**N (%)**	**N (%)**	
**Comorbidities**				
No. of chronic diseases, median (IQR)	4 (3, 6)	4.5 (3, 6)	4 (3, 5.8)	0.340^+^
No. of chronic diseases > 3	159 (66.8)	83 (72.2)	76 (61.8)	0.089^*^
Dyslipidemia	199 (83.6)	96 (83.5)	103 (83.7)	0.957^*^
Hypertension	201 (84.5)	101 (87.8)	100 (81.3)	0.165^*^
Diabetes mellitus	97 (40.8)	48 (41.7)	49 (39.8)	0.765^*^
Coronary artery disease	52 (21.8)	27 (23.5)	25 (20.3)	0.556^*^
Congestive heart failure	30 (12.6)	22 (19.1)	8 (6.5)	0.045^*^
Arrhythmia	54 (22.7)	27 (23.5)	27 (22.0)	0.779^*^
Dementia	6 (2.5)	5 (4.3)	1 (0.8)	0.110^*^
Cerebrovascular disease	35 (14.7)	22 (19.1)	13 (10.6)	0.062^*^
Hypothyroidism	8 (3.4)	3 (2.6)	5 (4.1)	0.723^*^
Hyperthyroidism	6 (2.5)	3 (2.6)	3 (2.4)	1.000^*^
Cirrhosis	13 (5.5)	6 (5.2)	7 (5.7)	0.872^*^
Chronic kidney disease	80 (33.6)	42 (36.5)	38 (30.9)	0.358^*^
Anemia	117 (49.2)	60 (52.2)	57 (46.3)	0.368^*^
Malignancy	39 (16.4)	15 (13.0)	24 (19.5)	0.178^*^
COPD	23 (9.7)	9 (7.8)	14 (11.4)	0.353^*^
Obstructive sleep apnea	5 (2.1)	4 (3.5)	1 (0.8)	0.200^*^

Data are presented as N (%), or median (interquartile range)

*Abbreviations: IQR* interquartile range, *COPD* chronic obstructive pulmonary disease

^*^Chi-square test

^+^Mann–Whitney U test

The hyperactive delirium group had a higher proportion of geriatric syndromes (*p* < 0.05) and significantly decreased functional capacities (*p* < 0.001), with greater tendencies for frailty (CFS score ≥ 5), malnutrition (NAF score > 5), depression (GDS-15 score > 5), impaired ADL (BADL score < 12 or Lawton IADL index < 8), and cognitive impairment (MoCA score < 25) (Supplementary Table 1). On physical examination, the number of patients with oxygen saturation < 95% was significantly higher in the hyperactive delirium group than in another group (*p* = 0.001), as shown in Supplementary Table 2.

The use of urinary catheters, NIMV, and physical restraints was significantly more frequent in patients with hyperactive delirium than those without delirium (*p* < 0.01), as presented in Supplementary Table 3. In Table 3, compared with the control group, the hyperactive delirium group had a significantly higher median number of prescribed medications per person [8 (IQR 6–11) vs. 7 (IQR 5–9.8), *p* = 0.028], particularly antipsychotics, analgesics, antidepressants, acetylcholinesterase inhibitors (AChEIs), and alpha-blockers (*p* < 0.05). However, there was no apparent difference in the prescriptions of muscle relaxants, corticosteroids, benzodiazepines, and antihistamines between the two groups. The biochemical parameters are revealed in Supplementary Table 4. The mean phosphate level and the median of partial thromboplastin time, prothrombin time, creatinine, alkaline phosphatase, and aspartate aminotransferase were substantially different between the two groups (*P* < 0.05).

**Table 3:** Prescribed medications among older medical inpatients stratified by exposure to hyperactive delirium

Medications	All (*n* = 238)	Hyperactive delirium (*n* = 115)	Control (*n* = 123)	*P-*value
		**N (%)**	**N (%)**	
Number of prescribed medications per person, median (IQR)	8 (5, 10)	8 (6, 11)	7 (5, 9.8)	0.028^+^
Polypharmacy	202 (84.9)	102 (88.7)	100 (81.3)	0.112^*^
Antihistamines	17 (7.1)	10 (8.7)	7 (5.7)	0.368^*^
Antimuscarinics	2 (0.8)	1 (0.9)	1 (0.8)	1.000^*^
Antipsychotics	8 (3.4)	8 (7.0)	0 (0.0)	0.003^*^
Skeletal muscle relaxant	2 (0.8)	1 (0.9)	1 (0.8)	1.000^*^
Benzodiazepines	25 (10.5)	11 (9.6)	14 (11.4)	0.648^*^
Corticosteroids	25 (10.5)	12 (10.4)	13 (10.6)	0.973^*^
Antiepileptics	10 (4.2)	7 (6.1)	3 (2.4)	0.204^*^
Analgesics	33 (13.9)	22 (19.1)	11 (8.9)	0.023^*^
Gabapenoids	22 (9.2)	15 (13.0)	7 (5.7)	0.050^*^
Opioids	7 (2.9)	3 (2.6)	4 (3.3)	1.000^*^
Antidepressants	19 (8.0)	15 (13.0)	4 (3.3)	0.005^*^
SSRIs	8 (3.4)	6 (5.2)	2 (1.6)	0.160^*^
Trazodone	3 (1.3)	3 (2.6)	0 (0.0)	0.111^*^
Mirtazapine	2 (0.8)	2 (1.7)	0 (0.0)	0.232^*^
Tricyclic antidepressants	6 (2.5)	3 (2.6)	3 (2.4)	1.000^*^
AChEIs	8 (3.4)	8 (7.0)	0 (0.0)	0.003^*^
Diuretics	67 (28.2)	36 (31.3)	31 (25.2)	0.296^*^
Alpha-blockers	14 (5.9)	11 (9.6)	3 (2.4)	0.020^*^
Antiarrhythmics	18 (7.6)	8 (7.0)	10 (8.1)	0.732^*^
Hypoglycemics	74 (31.1)	39 (33.9)	35 (28.5)	0.363^*^

Data are presented as N (%) or median (interquartile range)

*Abbreviations: IQR* interquartile range, *SSRIs* selective serotonin reuptake inhibitors, *AChEIs* acetylcholinesterase inhibitors

^*^Chi-square test

^+^Mann–Whitney U test

### Risk factors of hyperactive delirium

After univariate Cox proportional hazards analyses (Supplementary Table 5), multivariate Cox proportional hazards analyses were performed to explore independent risk factors for hyperactive delirium, as shown in Table 4. The independent risk factors for hyperactive delirium were CFS ≥ 5 (HR 2.79, 95% CI 1.69–4.62, *p* < 0.001), urinary incontinence (HR 1.69, 95%CI 1.11–2.57, *p* = 0.014) and MoCA score < 25 (HR 4.63, 95% CI 1.09–19.75, *p* = 0.038).

**Table 4:** Multivariate Cox proportional hazard for risk factors of hyperactive delirium in older medical inpatients

The risk factors	Multivariable model^a^	*p-*value
	**HR (95%CI)**	
CFS score ≥ 5	2.79 (1.69–4.62)	< 0.001*
MoCA score < 25	4.63 (1.09–19.75)	0.038
Urinary incontinence	1.69 (1.11–2.57)	0.014

Data are presented as hazard ratio (95% confidence interval)

*Abbreviations: CFS* Clinical Frailty Scale, *MoCA* Montreal Cognitive Assessment, *HR* hazard ratio, *CI* confidence interval

^a^ Covariate included in the adjusted model were the following: age, an education level (≤ 12 or > 12 yrs), Barthel ADL score (< 12 or ≥ 12), Lawton IADL score (< 8 or 8), MoCA score (< 25 or ≥ 25), NAF score (≤ 5 or > 5), GDS-15 score (≤ 5 or > 5), CFS score (< 5 or ≥ 5), number of prescribed medication, the history of antidepressants, analgesics, antipsychotics, and acetylcholinesterase inhibitors use, the history of congestive heart failure, urinary incontinence, fecal incontinence, and previous delirium, oxygen saturation < 95%, visual impairment, auditory impairment, the use of NIMV, physical restraint, and urinary catheter

^*^P < 0.01

### Characteristics of hyperactive delirium

As shown in Supplementary Table 6, the average onset of hyperactive delirium was 3 days (IQR 2–4) after admission. Most patients (71.3%) developed hyperactive delirium within 3 days after admission. The majority of older patients with hyperactive delirium (67%) had a RASS score of + 1. The incident rate for hyperactive delirium was 101.1 cases per 1000 person-days.

### Clinical outcomes of hyperactive delirium

**Fig. 2:**
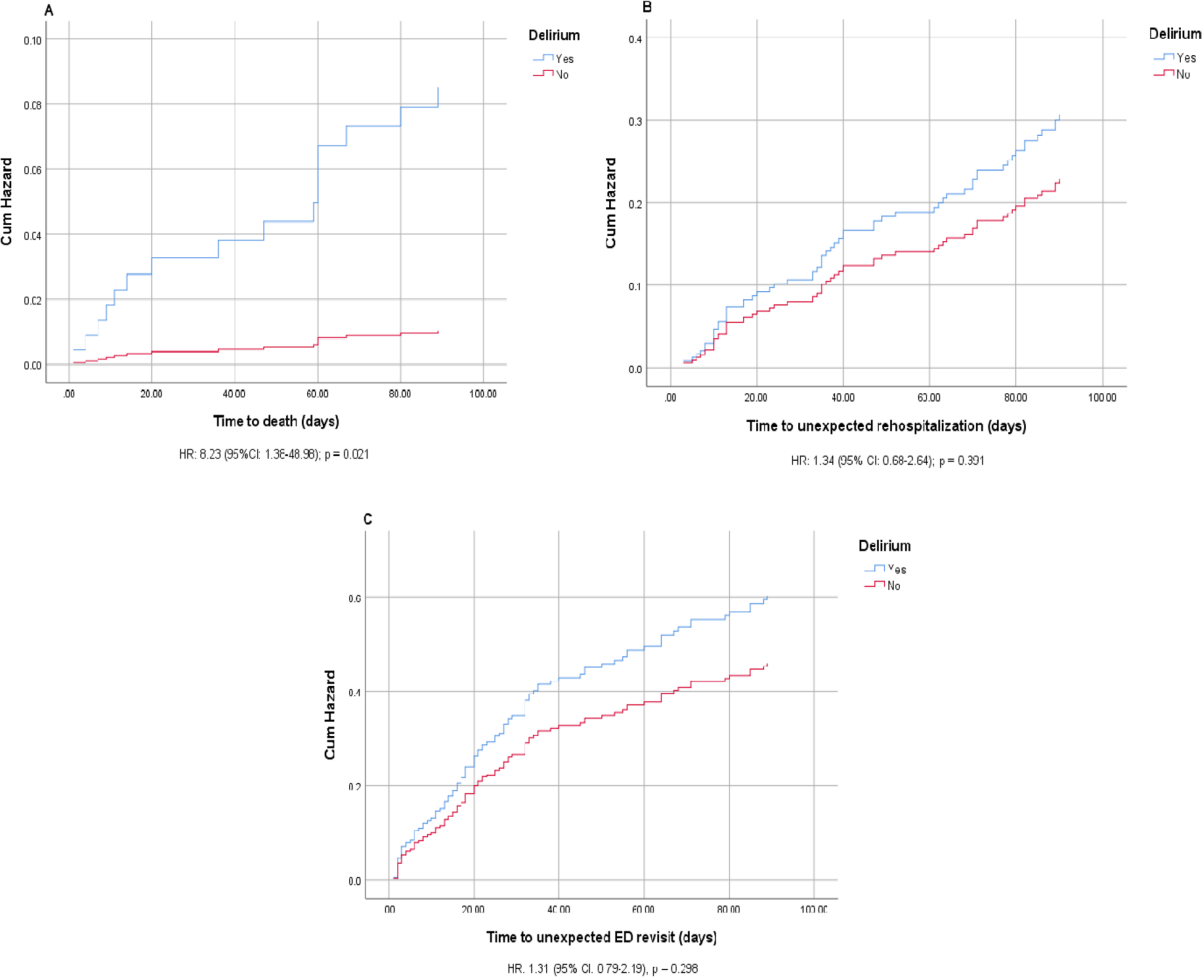
Multivariate Cox proportional hazard analysis of hyperactive delirium on clinical outcomes after discharge. **A** all-cause mortality (**B**) unexpected hospital readmission (**C**) unexpected ED revisit (adjusted by sex, age, polypharmacy, number of comorbidities, NAF score, MoCA score, CFS score)

The number of dead patients in the hyperactive delirium group was significantly higher than those in another group [12.2% (*n* = 14) vs. 1.6% (*n* = 2), *p* < 0.001] as well as unexpected ED visits and hospital readmissions (*p* < 0.05) at 90 days after discharge, as presented in Supplementary Table **7**. The 90-day clinical outcomes after discharge in both groups were analyzed using a multivariate Cox proportional hazard model, adjusted for potential confounders, including sex, age, polypharmacy, number of comorbidities, CFS score, NAF score, and MoCA score. In multivariate Cox proportional hazard analysis (Fig. 2), hyperactive delirium was independently associated with an 8-fold increase in 90-day mortality after discharge (HR 8.23, 95% CI 1.38–48.98, *p* = 0.021). However, patients with hyperactive delirium did not increase the risk of unexpected ED visits (HR 1.31, 95% CI 0.79–2.19, *p* = 0.298) and hospital readmission (HR 1.34, 95% CI 0.68–2.64, *p* = 0.391) during the 90-day follow-up after discharge.

## Discussion

This study explored risk factors and adverse clinical outcomes of hyperactive delirium in older medical inpatients. The novelty of the study is that it focused on hyperactive delirium in older medical inpatients aged 60 or older admitted to non-ICU wards. The four main findings were reported in the current study. First, approximately 48.3% of these older medical patients developed hyperactive delirium. Second, most patients who developed hyperactive delirium were admitted to medical non-ICU wards due to CHF and sepsis conditions. Third, urinary incontinence, cognitive impairment (MoCA score < 25), and frailty (CFS score ≥ 5) increased the risk of hyperactive delirium. Fourth, the occurrence of hyperactive delirium was a risk of death 90 days after discharge in this susceptible population.

The incidence of hyperactive delirium in this setting was 48.3%, higher than that reported by Inouye et al. [5]. and Khurana et al. [34], probably because of differences in delirium assessment tools. Apart from various screening tools, several studies found that medical practitioners often failed to detect delirium in 35%–65% of cases [35–37] due to the lack of conceptual understanding and the fluctuating manifestation of delirium [38].

In this study, sepsis and CHF were reported as leading causes of hospitalization in patients who developed hyperactive delirium, which is in line with recent studies [6, 34]. CHF can be a greater risk of delirium due to transient hemodynamic disturbance and cerebral hypoperfusion. Furthermore, medications with anticholinergic properties prescribed for the treatment of CHF, such as digoxin and furosemide, may be a cause of delirium [39, 40]. Sepsis-associated delirium is explained by the mechanisms of the combination of disturbance in cerebral perfusion, impaired cerebral glucose supply, and neuroinflammation [41, 42].

The current study reported a high prevalence of polypharmacy in this susceptible population. As stated in a previous study, polypharmacy was a related factor to delirium [11]. According to the 2023 Beers criteria [43], inappropriate medications such as anticholinergics and benzodiazepines may result in delirium and cognitive impairment [43]. In addition, certain antipsychotics and antidepressants have been found to precipitate delirium due to their potent antimuscarinic properties [43–45]. Therefore, physicians and pharmacists should implement thorough, comprehensive medication reviews and medication reconciliation to optimize prescribing practices for older medical inpatients in order to minimize the use of inappropriate medications associated with delirium [46, 47].

According to the systematic review by Ormseth et al., cognitive impairment (MoCA score < 25) and frailty (CFS score ≥ 5) were included in one of the identified 33 predisposing factors, and urinary incontinence was mentioned as a precipitating factor related to delirium [48]. The most common cause of cognitive impairment in the geriatric population is dementia. Delirium and dementia have a strongly complex inter-relationship owing to overlapping pathophysiology of both increased systemic inflammatory and neuroinflammatory biomarkers, leading to neuroaxonal damage [49]. The meta-analysis study of Persico et al. (8 studies,* n* = 5541) reported a 2.19-fold increased risk of delirium in individuals with frailty [50]. The common pathophysiological pathways between frailty and delirium are explained by cerebral metabolic insufficiency [51], vascular burden, and pro-inflammatory and neurodegenerative processes [52]. Urinary incontinence was one of the risk factors for hyperactive delirium in this study. Many older adults are prone to urge urinary incontinence. Antimuscarinics or anticholinergics are widely used to treat urge incontinence, resulting in blocking the action of acetylcholine neurotransmitters and delirium development [53].

Patients with hyperactive delirium had higher rates of 90-day all-cause mortality (12.2% vs. 1.6%; *p* = 0.001). This result is similar to preceding studies, which reported mortality rates after discharge ranging from 14 to 37% [54, 55]. Our study revealed that delirium was a significant risk of death after follow-up 90 days post-discharge (HR 8.23, 95% CI 1.38–48.98), which is consistent with the previous study [55]. According to the study of Cole et al., delirium persistence was found at 21% at six months post-discharge, and this finding may result in worsening cognitive and functional ability, increased morbidity, and mortality [56]. However, there was no significant increase in other outcomes, such as unexpected hospital readmission and ED visits in patients developing hyperactive delirium.

### Strengths and limitations

To the best of our knowledge, this was the first study that focused on the incidence, risk factors, and clinical outcomes of hyperactive delirium among older medical inpatients admitted to non-ICU wards in Thailand. The study's main strength was the comprehensive face-to-face interview and EMR review assessment. Trained medical assessors conducted the clinical and functional assessments. Furthermore, hyperactive delirium was assessed daily by trained medical staff using standardized diagnostic criteria, such as the CAM criteria and RASS score.

However, several limitations have to be taken into consideration. First, the study had a relatively modest sample size and a limited follow-up period. Second, some specific issues on hyperactive delirium, including symptom duration, severity, and number of events per patient, were not recorded. Third, the high prevalence of polypharmacy can influence both the development and assessment of hyperactive delirium. Furthermore, patient-specific factors, such as diverse population characteristics and multiple somatic comorbidities, may affect the onset of hyperactive delirium. Finally, although the multivariate Cox proportional hazard model was adjusted for numerous potential covariates, some unmeasured confounders might be missed.

### Further research and Implications

Among susceptible patients, a comprehensive history review and clinical assessment to determine risk factors, such as urinary incontinence, cognitive impairment (MoCA score < 25), and frailty (CFS ≥ 5), are warranted for early prediction and prevention of hyperactive delirium. Future prospective cohort studies need to cover longer follow-ups after discharge to explore further the significant adverse clinical outcomes of hyperactive delirium in hospitalized older patients.

## Conclusion

Among older medical inpatients admitted to non-ICU wards, the incidence rate of hyperactive delirium was 101.1 cases per 1000 person-days. Risk factors for hyperactive delirium included urinary incontinence, cognitive impairment, and being frail. Moreover, older patients with hyperactive delirium were found to have an increased risk of 90-day mortality after discharge.

## Supplementary Information

Additional file 1: Supplementary Assessment 1. Interview Questionnaire for Baseline Characteristics and Geriatric Syndrome. Supplementary Table 1. Geriatric conditions among older medical inpatients stratified by exposure to hyperactive delirium. Supplementary Table 2. Clinical measurements among older medical inpatients stratified by exposure to hyperactive delirium. Supplementary Table 3. The medical devices use among older medical inpatients stratified by exposure to hyperactive delirium. Supplementary Table 4. Biochemical profiles among older medical inpatients stratified by exposure to hyperactive delirium. Supplementary Table 5. Univariate Cox proportional hazard for risk factors of hyperactive delirium in older medical inpatients. Supplementary Table 6. The onset of hyperactive delirium after admission and RASS score among older medical inpatients. Supplementary Table 7. Adverse clinical outcomes of hyperactive delirium in older medical inpatients

## Data Availability

All data supporting our findings have been presented in the manuscript, and its supplementary information files. The datasets used and/or analyzed during the current study are available from the corresponding author upon reasonable request.

## Abbreviations

EMR: Electronic Medical Record
CFS: Clinical Frailty Scale
CAM: Confusion Assessment Method
DSM-V: Diagnostic and Statistical Manual of Mental Disorders, fifth edition
RASS: Richmond Agitation Sedation Scale
NAF: Nutritional Alert Form
MoCA: Montreal Cognitive Assessment
GDS: Geriatric Depression Scale
BMI: Body Mass Index
SBP: Systolic Blood Pressure
DBP: Diastolic Blood Pressure
LOS: Length of Hospital Stay
PR: Pulse Rate
BADL: Basic Activity of Daily Living
IADL: Instrumental Activity of Daily Living
ED: Emergency Department
OPD: Out Patient Department
CHF: Congestive Heart Failure
COPD: Chronic Obstructive Pulmonary Disease

## References

[1] World Health Organization. World Report on Ageing and Health; World Health Organization: Geneva, Switzerland, 2015. Available online: http://www.who.int/ageing/events/world-report-2015-launch. Accessed 12 Jan 2024.

[2] Foundation of Thai Gerontology Research and Development institute (TGRI). Situation of the Thai older persons 2021. Nakhon Pathom: Institute for Population and Social Research, Mahidol University; 2021. 120 p.

[3] He Z, Bian J, Carretta HJ, Lee J, Hogan WR, Shenkman E, et al. Prevalence of multiple chronic conditions among older adults in Florida and the United States: comparative analysis of the OneFlorida data trust and national inpatient sample. J Med Internet Res. 2018;20(4): e137. 29650502 10.2196/jmir.8961 PMC5920146

[4] Cornwell J, Levenson R, Sonola L, Poteliakhoff E. Continuity of care for older hospital patients: a call for action; 2012. https://www.kingsfund.org.uk/publications/continuity-care-older-hospital-patients. Accessed 12 Jan 2024.

[5] Inouye SK, Westendorp RG, Saczynski JS. Delirium in elderly people. Lancet. 2014;383(9920):911–22. 23992774 10.1016/S0140-6736(13)60688-1 PMC4120864

[6] Zhang M, Zhang X, Gao L, Yue J, Jiang X. Incidence, predictors and health outcomes of delirium in very old hospitalized patients: a prospective cohort study. BMC Geriatr. 2022;22(1):262. 35351018 10.1186/s12877-022-02932-9 PMC8966247

[7] Li X, Zhang L, Gong F, Ai Y. Incidence and risk factors for delirium in older patients following intensive care unit admission: a prospective observational study. J Nurs Res. 2020;28(4): e101. 32692119 10.1097/jnr.0000000000000384

[8] American Psychiatric Association. Diagnostic and statistical manual of mental disorders (DSM-5). 5th ed. Washington DC: American Psychiatric Association; 2013.

[9] Bellelli G, Morandi A, Trabucchi M, Caironi G, Coen D, Fraticelli C, et al. Italian intersociety consensus on prevention, diagnosis, and treatment of delirium in hospitalized older persons. Intern Emerg Med. 2018;13(1):113–21. 28741278 10.1007/s11739-017-1705-x

[10] Srinonprasert V, Pakdeewongse S, Assanasen J, Eiamjinnasuwat W, Sirisuwat A, Limmathuroskul D, et al. Risk factors for developing delirium in older patients admitted to general medical wards. J Med Assoc Thai. 2011;94(2):99. 21721434

[11] Kurisu K, Miyabe D, Furukawa Y, Shibayama O, Yoshiuchi K. Association between polypharmacy and the persistence of delirium: a retrospective cohort study. Biopsychosoc Med. 2020;14:25. 33042216 10.1186/s13030-020-00199-3 PMC7541288

[12] Stuhec M. Antipsychotic treatment in elderly patients on polypharmacy with schizophrenia. Curr Opin Psychiatry. 2022;35(5):332–7. 35788124 10.1097/YCO.0000000000000808

[13] Becker MH. The health belief model and personal health behavior. Health Educ Monogr. 1974;2:324–473.

[14] Inouye SK, van Dyck CH, Alessi CA, Balkin S, Siegal AP, Horwitz RI. Clarifying confusion: the confusion assessment method. A new method for detection of delirium. Ann Intern Med. 1990;113(12):941–8. 2240918 10.7326/0003-4819-113-12-941

[15] Evensen S, Hylen Ranhoff A, Lydersen S, Saltvedt I. The delirium screening tool 4AT in routine clinical practice: prediction of mortality, sensitivity and specificity. Eur Geriatr Med. 2021;12(4):793–800. 33813725 10.1007/s41999-021-00489-1 PMC8321971

[16] Gual N, Inzitari M, Carrizo G, Calle A, Udina C, Yuste A, et al. Delirium subtypes and associated characteristics in older patients with exacerbation of chronic conditions. Am J Geriatr Psychiatry. 2018;26(12):1204–12. 30131288 10.1016/j.jagp.2018.07.003

[17] Kinchin I, Mitchell E, Agar M, Trépel D. The economic cost of delirium: a systematic review and quality assessment. Alzheimers Dement. 2021;17(6):1026–41. 33480183 10.1002/alz.12262

[18] Ostuzzi G, Gastaldon C, Papola D, Fagiolini A, Dursun S, Taylor D, et al. Pharmacological treatment of hyperactive delirium in people with COVID-19: rethinking conventional approaches. Ther Adv Psychopharmacol. 2020;10:2045125320942703. 32733668 10.1177/2045125320942703 PMC7372613

[19] Huang X, Lin J, Demner-Fushman D. Evaluation of PICO as a knowledge representation for clinical questions. AMIA Annu Symp Proc. 2006;2006:359–63. 17238363 PMC1839740

[20] Ngamjarus C. n4Studies: sample size calculation for an epidemiological study on a smart device. Siriraj Med J. 2016;68(3):160–70.

[21] Ely EW, Truman B, Shintani A, Thomason JW, Wheeler AP, et al. Monitoring sedation status over time in ICU patients: reliability and validity of the Richmond Agitation-Sedation Scale (RASS). JAMA. 2003;289:2983–91. 12799407 10.1001/jama.289.22.2983

[22] Pasina L, Colzani L, Cortesi L, Tettamanti M, Zambon A, Nobili A, et al. Relation between delirium and anticholinergic drug burden in a cohort of hospitalized older patients: an observational study. Drugs Aging. 2019;36(1):85–91. 30484239 10.1007/s40266-018-0612-9

[23] Fi M. Functional evaluation: the Barthel index. Md State Med J. 1965;14:61–5. 14258950

[24] Lawton MP, Brody EM. Assessment of older people: self-maintaining and instrumental activities of daily living. Gerontologist. 1969;9(3):179–86. 5349366

[25] Nasreddine ZS, Phillips NA, Bédirian V, Charbonneau S, Whitehead V, Collin I, et al. The montreal cognitive assessment, MoCA: a brief screening tool for mild cognitive impairment. J Am Geriatr Soc. 2005;53(4):695–9. 15817019 10.1111/j.1532-5415.2005.53221.x

[26] Tangwongchai S, Phanasathit M, Charernboon T, Akkayagorn L, Hemrungrojn S, Phanthumchinda K, et al. The validity of Thai version of The Montreal Cognitive Assessment (MoCA-T). Dement Neuropsychol. 2009;3:136–78.

[27] Poon LW, Crook TE, Davis KL, Eisdorfer CE, Gurland BJ, Kaszniak AW, Thompson LW. Handbook for clinical memory assessment of older adults. American Psychological Association; 1986.

[28] Nuttall FQ. Body mass index: obesity, BMI, and health: a critical review. Nutr Today. 2015;50(3):117–28. 27340299 10.1097/NT.0000000000000092 PMC4890841

[29] Komindrg S, Tangsermwong T, Janepanish P. Simplified malnutrition tool for Thai patients. Asia Pac J Clin Nutr. 2013;22(4):516–21. 24231010 10.6133/apjcn.2013.22.4.06

[30] Church S, Rogers E, Rockwood K, Theou O. A scoping review of the clinical frailty scale. BMC Geriatr. 2020;20(1):393. 33028215 10.1186/s12877-020-01801-7 PMC7540438

[31] Rockwood K, Song X, MacKnight C, Bergman H, Hogan DB, McDowell I, et al. A global clinical measure of fitness and frailty in elderly people. CMAJ. 2005;173(5):489–95. 16129869 10.1503/cmaj.050051 PMC1188185

[32] Ramírez Echeverría MdL, Schoo C, Paul M. Delirium. [Updated 2022 Nov 19]. In: StatPearls. Treasure Island (FL): StatPearls Publishing; 2024. https://www.ncbi.nlm.nih.gov/books/NBK470399/. Accessed 12 Jan 2024.

[33] Charoensak S, Thunmanurukkit A, Sittironnarit G, Sartra T. Validity and reliability of the Thai version of the confusion assessment method. J Med Assoc Thai. 2014;97(1):113–7. 24701738

[34] Khurana V, Gambhir IS, Kishore D. Evaluation of delirium in elderly: a hospital-based study. Geriatr Gerontol Int. 2011;11(4):467–73. 21592270 10.1111/j.1447-0594.2011.00710.x

[35] Al Farsi RS, Al Alawi AM, Al Huraizi AR, Al-Saadi T, Al-Hamadani N, Al Zeedy K, et al. Delirium in medically hospitalized patients: prevalence, recognition and risk factors: a prospective cohort study. J Clin Med. 2023;12(12): 3897. 37373591 10.3390/jcm12123897 PMC10299512

[36] Zalon ML, Sandhaus S, Kovaleski M, Roe-Prior P. Hospitalized older adults with established Delirium: recognition, documentation, and reporting. J Gerontol Nurs. 2017;43(3):32–40. 27845806 10.3928/00989134-20161109-01

[37] Praditsuwan R, Srinonprasert V. Unrecognized delirium is prevalent among older patients Admitted to general medical wards and Lead to higher mortality rate. J Med Assoc Thai. 2016;99(8):904–12. 29947497

[38] El Hussein M, Hirst S, Salyers V. Factors that contribute to underrecognition of delirium by registered nurses in acute care settings: a scoping review of the literature to explain this phenomenon. J Clin Nurs. 2015;24(7–8):906–15. 25293502 10.1111/jocn.12693

[39] Ampadu J, Morley JE. Heart failure and cognitive dysfunction. Int J Cardiol. 2015;178:12–23. 25464210 10.1016/j.ijcard.2014.10.087

[40] Morley JE. Anticholinergic medications and cognition. J Am Med Dir Assoc. 2011;12(8):543-543.e1. 21856240 10.1016/j.jamda.2011.07.008

[41] Atterton B, Paulino MC, Povoa P, Martin-Loeches I. Sepsis associated delirium. Medicina (Kaunas). 2020;56(5): 240. 32443606 10.3390/medicina56050240 PMC7279289

[42] Tokuda R, Nakamura K, Takatani Y, Tanaka C, Kondo Y, Ohbe H, et al. Sepsis-associated delirium: a narrative review. J Clin Med. 2023;12(4): 1273. 36835809 10.3390/jcm12041273 PMC9962483

[43] By the 2023 American Geriatrics Society Beers Criteria® Update Expert Panel. American geriatrics society 2023 updated AGS beers criteria® for potentially inappropriate medication use in older adults. J Am Geriatr Soc. 2023;71(7):2052–81. 37139824 10.1111/jgs.18372 PMC12478568

[44] Gerretsen P, Pollock BG. Drugs with anticholinergic properties: a current perspective on use and safety. Expert Opin Drug Saf. 2011;10(5):751–65. 21635190 10.1517/14740338.2011.579899

[45] Gray SL, Hanlon JT. Anticholinergic medication use and dementia: latest evidence and clinical implications. Ther Adv Drug Saf. 2016;7(5):217–24. 27695623 10.1177/2042098616658399 PMC5014048

[46] Stuhec M, Batinic B. Clinical pharmacist interventions in the transition of care in a mental health hospital: case reports focused on the medication reconciliation process. Front Psychiatry. 2023;14: 1263464. 38205081 10.3389/fpsyt.2023.1263464 PMC10777203

[47] Stuhec M, Hahn M, Taskova I, Bayraktar I, Fitzgerald I, Molitschnig L, et al. Clinical pharmacy services in mental health in Europe: a commentary paper of the European society of clinical pharmacy special interest group on mental health. Int J Clin Pharm. 2023;45(5):1286–92. 37755642 10.1007/s11096-023-01643-4 PMC10600282

[48] Ormseth CH, LaHue SC, Oldham MA, Josephson SA, Whitaker E, Douglas VC. Predisposing and Precipitating Factors Associated With Delirium: A Systematic Review. JAMA Netw Open. 2023;6(1): e2249950. 36607634 10.1001/jamanetworkopen.2022.49950 PMC9856673

[49] Fong TG, Inouye SK. The inter-relationship between delirium and dementia: the importance of delirium prevention. Nat Rev Neurol. 2022;18(10):579–96. 36028563 10.1038/s41582-022-00698-7 PMC9415264

[50] Persico I, Cesari M, Morandi A, Haas J, Mazzola P, Zambon A, et al. Frailty and delirium in older adults: a systematic review and meta-analysis of the literature. J Am Geriatr Soc. 2018;66:2022–30. 30238970 10.1111/jgs.15503

[51] Wilson JE, Mart MF, Cunningham C, Shehabi Y, Girard TD, MacLullich AJM, et al. Delirium. Nat Rev Dis Primer. 2020;6(1):90. 10.1038/s41572-020-00223-4 PMC9012267 33184265

[52] Xu Y, Wang M, Chen D, Jiang X, Xiong Z. Inflammatory biomarkers in older adults with frailty: a systematic review and meta-analysis of cross-sectional studies. Aging Clin Exp Res. 2022;34:971–87. 34981430 10.1007/s40520-021-02022-7

[53] Hogan DB. Revisiting the O complex: urinary incontinence, delirium and polypharmacy in elderly patients. CMAJ. 1997;157(8):1071–7. 9347778 PMC1228263

[54] Siddiqi N, House AO, Holmes JD. Occurrence and outcome of delirium in medical inpatients: a systematic literature review. Age Ageing. 2006;35:350–64. 16648149 10.1093/ageing/afl005

[55] Al Huraizi AR, Al-Maqbali JS, Al Farsi RS, Al Zeedy K, Al-Saadi T, Al-Hamadani N, Al Alawi AM. Delirium and its association with short- and long-term health outcomes in medically admitted patients: a prospective study. J Clin Med. 2023;12(16): 5346. 37629388 10.3390/jcm12165346 PMC10455146

[56] Cole MG, Ciampi A, Belzile E, Zhong L. Persistent delirium in older hospital patients: a systematic review of frequency and prognosis. Age Ageing. 2009;38(1):19–26. 19017678 10.1093/ageing/afn253

