# Supplementary material for: Risk factors and outcomes of hyperactive delirium in older medical inpatients admitted to non-intensive care unit: a prospective cohort study
Source: BMC Psychiatry. 2025 Apr 3;25:330. doi: 10.1186/s12888-025-06731-5 (PMC11969751; doi:10.1186/s12888-025-06731-5)
Supplement: Supplementary file 1 — Additional file 1: Supplementary Assessment 1. Interview Questionnaire for Baseline Characteristics and Geriatric Syndrome. Supplementary Table 1. Geriatric conditions among older medical inpatients stratified by exposure to hyperactive delirium. Supplementary Table 2. Clinical measurements among older medical inpatients stratified by exposure to hyperactive delirium. Supplementary Table 3. The medical devices use among older medical inpatients stratified by exposure to hyperactive delirium. Supplementary Table 4. Biochemical profiles among older medical inpatients stratified by exposure to hyperactive delirium. Supplementary Table 5. Univariate Cox proportional hazard for risk factors of hyperactive delirium in older medical inpatients. Supplementary Table 6. The onset of hyperactive delirium after admission and RASS score among older medical inpatients. Supplementary Table 7. Adverse clinical outcomes of hyperactive delirium in older medical inpatients [file 12888_2025_6731_MOESM1_ESM.zip › Supplementary Assessment 1. Interview Questionnaire for Baseline Characteristics and Geriatric Syndrome.pdf]

ID number ________________

Supplementary Assessment 1. Interview Questionnaire for Baseline Characteristics and Geriatric Syndrome

Part 1: Baseline Characteristics	
Married status	1. Married 2. Single 3. Widowed<br>4. Separated 5. Others..............
BW (kgs)	…………………………
Ht (cms)	…………………………
Education	1.<6 years 2. 6-12 years 3. >12 years
Primary Caregiver	1 Self-care 2. Spouse 3. Daughter/son<br>4. Sibling 5. Parents 6. Others............
Drug administrator	1 Self-care 2. Spouse 3. Daughter/son<br>4. Sibling 5. Parents 6. Others............
No.Care drug	1. 1 person 2. 2 persons 3. ≥ 3 persons
Living	1. Home with help 2. Home care 3. Hospital 4. Home
Discharged to	1. Home 2. Home care 3. Death<br>4. Home with help 5. Hospital
Type of discontinuation	1. Death 2. Transfer to ICU 3. Discharge
Living alone	1. Yes 2. No
Smoking	1. Yes 2. No
Drinking	1. Yes 2. No
Substance abuse	1. Yes 2. No
Herbal use	1. Yes 2. No
<u>Geriatric syndrome</u>	
Urinary incontinence	1. Yes 2. No
Fecal incontinence	1. Yes 2. No
Previous delirium	1. Yes 2. No
Pressure ulcer	1. Yes 2. No

Visual impairment	1. Yes 2. No
Auditory impairment	1. Yes 2. No
Previous Fall in 3 months	1. Yes (/y) 2. No
Significant weight loss<br>(5% in 1 mo, 10% in 6 mo)	1. Yes 2. No
Pain	1. Yes 2. No
Ambulation	1. Wheelchair 2. Walk 3. Walk with support<br>4. Bedbound 5.Bedridden
