# Supplementary material for: Risk factors and outcomes of hyperactive delirium in older medical inpatients admitted to non-intensive care unit: a prospective cohort study
Source: BMC Psychiatry. 2025 Apr 3;25:330. doi: 10.1186/s12888-025-06731-5 (PMC11969751; doi:10.1186/s12888-025-06731-5)
Supplement: Supplementary file 1 — Additional file 1: Supplementary Assessment 1. Interview Questionnaire for Baseline Characteristics and Geriatric Syndrome. Supplementary Table 1. Geriatric conditions among older medical inpatients stratified by exposure to hyperactive delirium. Supplementary Table 2. Clinical measurements among older medical inpatients stratified by exposure to hyperactive delirium. Supplementary Table 3. The medical devices use among older medical inpatients stratified by exposure to hyperactive delirium. Supplementary Table 4. Biochemical profiles among older medical inpatients stratified by exposure to hyperactive delirium. Supplementary Table 5. Univariate Cox proportional hazard for risk factors of hyperactive delirium in older medical inpatients. Supplementary Table 6. The onset of hyperactive delirium after admission and RASS score among older medical inpatients. Supplementary Table 7. Adverse clinical outcomes of hyperactive delirium in older medical inpatients [file 12888_2025_6731_MOESM1_ESM.zip › Supplementary Table 5.docx]

**Supplementary Table 5. Univariate Cox proportional hazard for risk factors of hyperactive delirium in older medical inpatients**

| **The risk factors** | **Univariable model** | **p-value** |
| --- | --- | --- |
|  | **HR (95%CI)** |  |
| Age | 1.06 (1.04–1.09) | <0.001 |
| Educational level ≤12 years | 1.65 (1.03–2.66) | 0.037 |
| History of congestive heart failure | 0.45 (0.18–1.11) | 0.081 |
| Urinary incontinence | 2.07 (1.43–2.99) | <0.001 |
| Fecal incontinence | 2.02 (1.14–3.63) | 0.016 |
| Visual impairment | 2.34 (1.65–3.39) | <0.001 |
| Auditory impairment | 2.63 (1.62–4.29) | <0.001 |
| History of previous delirium | 2.39 (1.16–4.93) | 0.018 |
| Oxygen saturation < 95% | 1.86 (1.19–2.89) | 0.006 |
| MoCA score <25 | 11.44 (2.83–46.31) | 0.001 |
| CFS score ≥ 5 | 4.32 (2.82–6.62) | <0.001 |
| NAF score > 5 | 3.17 (1.78–5.64) | <0.001 |
| GDS-15 score >5 | 3.46 (2.38–5.03) | <0.001 |
| BADL score <12 | 2.49 (1.71–3.63) | <0.001 |
| IADL score <8 | 7.27 (2.68–19.73) | <0.001 |
| Urinary catheter | 1.89 (1.28–2.78) | 0.001 |
| Non-invasive mechanical ventilation | 1.72 (1.05–2.81) | 0.031 |
| Physical restraint | 2.68 (1.46–4.89) | 0.001 |
| Number of prescribed medications | 1.05 (1.00–1.09) | 0.043 |
| Antipsychotics use | 4.16 (2.00–8.64) | <0.001 |
| Analgesics use | 1.49 (0.93–2.38) | 0.090 |
| Antidepressants use | 2.09 (1.21–3.61) | 0.008 |
| Acetylcholinesterase inhibitors use | 3.03 (1.46–6.27) | 0.003 |

**Data are presented as** hazard ratio (95% confidence interval)

**Abbreviations:** CFS, Clinical Frailty Scale; MoCA, Montreal Cognitive Assessment; NAF, Nutritional Alert Form; GDS-15, Geriatric Depression Scale-15; BADL, Basic activities of daily living; IADL, Instrumental activities of daily living; HR, hazard ratio; CI, confidence interval
