# Supplementary material for: Risk factors and outcomes of hyperactive delirium in older medical inpatients admitted to non-intensive care unit: a prospective cohort study
Source: BMC Psychiatry. 2025 Apr 3;25:330. doi: 10.1186/s12888-025-06731-5 (PMC11969751; doi:10.1186/s12888-025-06731-5)
Supplement: Supplementary file 1 — Additional file 1: Supplementary Assessment 1. Interview Questionnaire for Baseline Characteristics and Geriatric Syndrome. Supplementary Table 1. Geriatric conditions among older medical inpatients stratified by exposure to hyperactive delirium. Supplementary Table 2. Clinical measurements among older medical inpatients stratified by exposure to hyperactive delirium. Supplementary Table 3. The medical devices use among older medical inpatients stratified by exposure to hyperactive delirium. Supplementary Table 4. Biochemical profiles among older medical inpatients stratified by exposure to hyperactive delirium. Supplementary Table 5. Univariate Cox proportional hazard for risk factors of hyperactive delirium in older medical inpatients. Supplementary Table 6. The onset of hyperactive delirium after admission and RASS score among older medical inpatients. Supplementary Table 7. Adverse clinical outcomes of hyperactive delirium in older medical inpatients [file 12888_2025_6731_MOESM1_ESM.zip › Supplementary Table 7.doc]

**Supplementary Table 7. Adverse clinical outcomes of hyperactive delirium in older medical inpatients**

| **Adverse clinical outcomes** | **All (n=238)** | **Hyperactive delirium (n=115)** | **Control (n=123)** | **P-value** |
| --- | --- | --- | --- | --- |
|  | **N (%)** | **N (%)** | **N (%)** |  |
| **30-day post discharge** | | | | |
| Mortality | 8 (3.4) | 7 (6.1) | 1 (0.8) | 0.031 |
| Unexpected hospital readmission | 26 (10.9) | 16 (13.9) | 10 (8.1) | 0.153 |
| Unexpected ED revisit | 65 (27.3) | 35 (30.4) | 30 (24.4) | 0.296 |
| **90-day post discharge** | | | | |
| Mortality | 16 (6.7) | 14 (12.2) | 2 (1.6) | 0.001^*^ |
| Unexpected hospital readmission | 61 (25.6) | 37 (32.2) | 24 (19.5) | 0.025 |
| Unexpected ED revisit | 99 (42.6) | 57 (49.6) | 42 (34.1) | 0.016 |

^*^ p <0.01

**Data are presented as** N (%)

**Abbreviations:** ED, emergency department
